# *FOXO3* longevity genotype mitigates the increased mortality risk in men with a cardiometabolic disease

**DOI:** 10.18632/aging.202175

**Published:** 2020-12-01

**Authors:** Randi Chen, Brian J. Morris, Timothy A. Donlon, Kamal H. Masaki, D. Craig Willcox, Philip M.C. Davy, Richard C. Allsopp, Bradley J. Willcox

**Affiliations:** 1 Department of Research, Kuakini Medical Center, Honolulu, HI 96817, USA; 2 Department of Geriatric Medicine, John A. Burns School of Medicine, University of Hawaii, Honolulu, HI 96813, USA; 3 School of Medical Sciences, University of Sydney, Sydney, New South Wales, Australia; 4 Department of Cell and Molecular Biology, John A. Burns School of Medicine, University of Hawaii, Honolulu, HI 96813, USA; 5 Institute for Biogenesis Research, University of Hawaii, Honolulu, HI 96822, USA; 6 Department of Pathology, John A. Burns School of Medicine, University of Hawaii, Honolulu, HI 96813, USA; 7 Department of Human Welfare, Okinawa International University, Ginowan, Okinawa, Japan

**Keywords:** longevity, genetics, mortality, FOXO3, resilience

## Abstract

*FOXO3* is a prominent longevity gene. To date, no-one has examined whether longevity-associated *FOXO3* genetic variants protect against mortality in all individuals, or only in those with aging-related diseases. We therefore tested longevity-associated *FOXO3* single nucleotide polymorphisms in a haplotype block for association with mortality in 3,584 elderly American men of Japanese ancestry, 2,512 with and 1,072 without a cardiometabolic disease (CMD). At baseline (1991–1993), 1,010 CMD subjects had diabetes, 1,919 had hypertension, and 738 had coronary heart disease (CHD). Follow-up until Dec 31, 2019 found that in CMD-affected individuals, longevity-associated alleles of *FOXO3* were associated with significantly longer lifespan: haplotype hazard ratio 0.81 (95% CI 0.72-0.91; diabetes 0.77, hypertension 0.82, CHD 0.83). Overall, men with a CMD had higher mortality than men without a CMD (*P*=6x10^-7^). However, those men with a CMD who had the *FOXO3* longevity genotype had similar survival as men without a CMD. In men without a CMD there was no association of longevity-associated alleles of *FOXO3* with lifespan. Our study provides novel insights into the basis for the long-established role of *FOXO3* as a longevity gene. We suggest that the *FOXO3* longevity genotype increases lifespan only in at-risk individuals by protection against cardiometabolic stress.

## INTRODUCTION

Longevity can be accompanied by either healthy aging (i.e., an absence of life-threatening diseases and chronic conditions), or unhealthy aging, in which elderly individuals live long lives with chronic conditions. Genetic factors make a 15–40% contribution to lifespan [1], and may be as high as 48% in the oldest-old [2]. At very old age (> 90 years) specific longevity genes may dominate over environmental influences in determination of eventual lifespan, with evidence that there is a stronger effect in males [3].

Our study focused on *FOXO3*, the gene encoding the forkhead/winged helix box-O3 (FoxO3) transcription factor. Minor alleles of single nucleotide polymorphisms (SNPs) located in *FOXO3* have been found to be strongly associated with human longevity in multiple population subgroups worldwide [4–6]. This includes genome-wide association study data [7]. We found previously that a haplotype comprised of 14 *FOXO3* SNPs is associated with increased likelihood of living close to 100 years in the Kuakini Honolulu Heart Program/ Kuakini and Honolulu-Asia Aging Study (KHHP/KHAAS) cohort [8]. FoxO3 can differentially induce gene expression programs depending on chromatin architecture by binding to specific enhancers, activating these by causing changes in histone acetylation, and subsequent recruitment of RNA polymerase II [9]. In response to cellular stress, *FOXO3* may also function at the genomic level by facilitating long-range gene-gene interactions, changes in chromatin conformation, and interaction of topologically associated domains to regulate multiple neighboring genes involved in various processes that contribute to cell resilience, namely autophagy, stress response, energy/nutrient sensing, cell proliferation, apoptosis, and stem cell maintenance [8, 10]. The expression of at least some of these neighboring genes is increased following cellular stress (see supplement to ref [8]). Thus, *FOXO3* is positioned at the center of an “aging hub.” The longevity alleles of *FOXO3* enable enhanced *FOXO3* expression, thereby activating other genes in its functional neighborhood. Physiologically, we now know that the major reason for the increase in lifespan conferred by protective alleles of *FOXO3* is protection against death from coronary heart disease (CHD) [11], although in other cohorts such protection may extend to cancer and stroke [12, 13].

The present study addresses the hypothesis that the *FOXO3* longevity-associated alleles increase cell and organism resilience by protecting against cellular stress caused by chronic conditions of aging. To test this hypothesis, we compared the effect of longevity-associated alleles with lifespan in subjects with and without type 2 diabetes, CHD and hypertension (collectively referred to as cardiometabolic diseases, CMDs).

## RESULTS

### Characteristics of subjects

Supplementary Table 1 shows age-adjusted baseline (1991–1993) characteristics of men in the study, adjusting for age, genotypes of the strongest SNP (*rs2802292*), and prevalence of medical conditions. By 31 December 2019, 3,548 out of 3,584 of the subjects had died during the 29 years of follow-up from exam 4. At exam 4 (baseline) among the 3,584 participants, there were 21% who had been diagnosed with CHD, 29% with diabetes, 54% with hypertension, and 14% with cancer. Seventy percent had at least one CMD, and 5.2% had all 3 components of CMD. Mean age at death was 88.6 ± 6.1 years for those with at least one of these CMDs, and 89.5 ± 6.0 years for those who did not have any CMD (p < 0.0001).

### *FOXO3* genotype and survival in CMD and non-CMD subjects.

**Figure 1:**
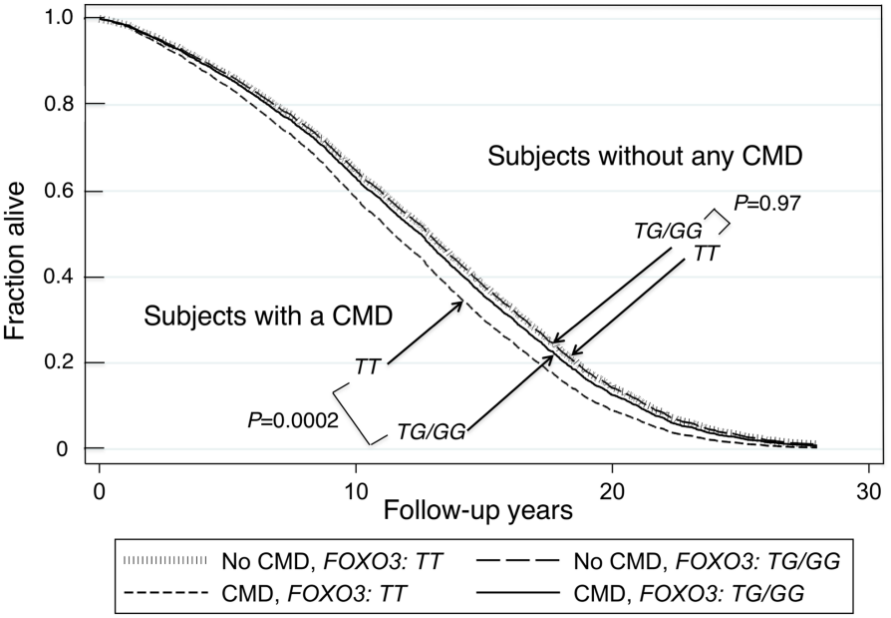
**Survival curves spanning the period from baseline (1991–1993) to Dec 31, 2019 for subjects with and without a CMD according to whether they were carriers of the longevity-associated *G* allele of SNP *rs2802292*.** The survival probabilities were estimated from the Cox proportional hazard model (see Methods) h(t) = h(t0) * exp(β1*Age + β2*BMI + β3*Glucose + β4*CMD + β5*FOXO3_G + β6* (CMD*FOXO3_G)) by fixing age at 75 years, BMI at the mean, 23.5 kg/m^2^, and glucose at the mean, 113 mg/dL (where β6 is the effect of the interaction of CMD with *FOXO3* genotype (*G* carriers vs. *TT* genotype) on mortality, giving *P*(β6) = 0.04). The *P* values for comparison of survival curves for the group without any CMD for *FOXO3-G* carriers vs. *FOXO3-TT*, and comparison of survival curves for the group with a CMD for *FOXO3-G* carriers vs. *FOXO3-TT*, were *P*=0.97 and *P*=0.0002, respectively. The *P*-values for comparison of survival curves for *FOXO3-TT* carriers or for *FOXO3-G* carriers for those with a CMD versus those without any CMD, were *P*=0.000039 and *P*=0.28 respectively.

**Figure 2:**
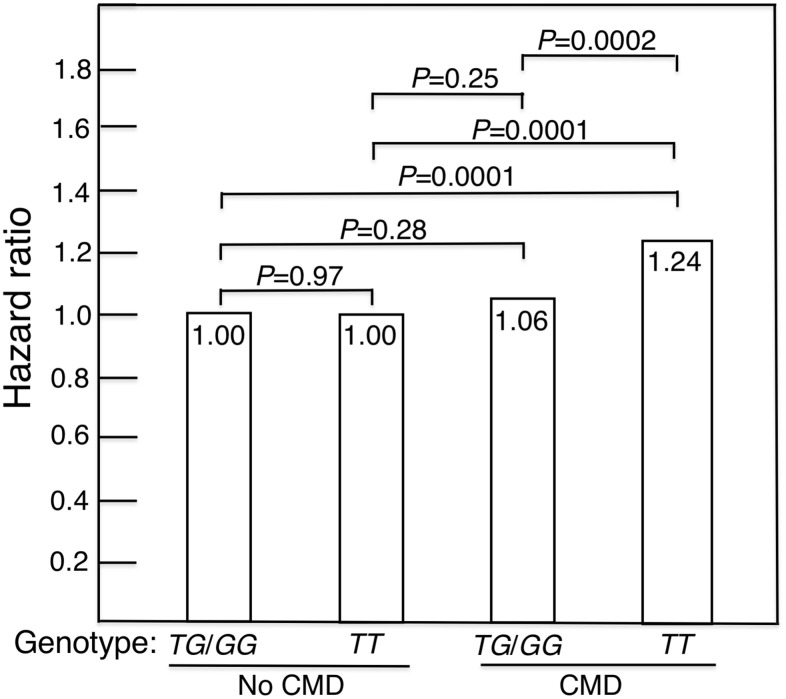
**Mortality risk expressed as hazard ratio, adjusted for age, BMI and glucose, for CMD subjects and subjects without a CMD according to whether they were carriers of the longevity-associated (*G*) allele of the *FOXO3* SNP *rs2802292* (*TG*/*GG*) or were homozygous for the major (*T*) allele (i.e., were *TT*).** In men with CMD and the *FOXO3* longevity genotype, mortality risk was reduced to normal (i.e., did not differ significantly from that of men without CMD).

The 14 *FOXO3* SNPs (shown in Supplementary Figures 1, 2) likely function as a *cis*-regulatory unit. Survival curves for men without a CMD and those with a CMD showed that the former lived longer (Kaplan-Meier Log-rank χ^2^ = 24.8, p = 6.4x10^-7^). Figure 1 shows survival curves for CMD subjects and subjects without a CMD according to whether they were carriers of the longevity-associated (*G*) allele of the strongest *FOXO3* SNP, *rs2802292*, or were homozygous for the major (*T*) allele. These curves were determined using a Cox proportional hazard model adding an interaction term of *FOXO3* with CMD. Only in CMD subjects was the *G* allele associated with greater lifespan than *TT* (p = 0.0002). Subjects without a CMD had the longest lifespans. Moreover, in contrast to CMD subjects, *FOXO3* genotype was not associated with lifespan in subjects without a CMD. Figure 2 shows mortality risk as hazard ratio for CMD subjects and subjects without a CMD according to whether they were carriers of the longevity-associated (*G*) allele of the strongest *FOXO3* SNP, *rs2802292*, or were homozygous for the major (*T*) allele. In men with CMD and the *FOXO3* longevity genotype, mortality was reduced to that of men without CMD.

Table 1 shows age-adjusted baseline characteristics of men with CMD and men without a CMD. Analyses found no evidence of population stratification in the dataset (data not shown). In subjects with *either* diabetes, CHD, or hypertension (CMD) *at baseline*, *G* allele carriers had significantly lower CHD prevalence, but higher diabetes and hypertension (Table 1). In CMD subjects, after adjusting for co-variates, minor allele carriers of each of the 8 *FOXO3* SNPs tested exhibited an association with longer lifespan (Table 2). In contrast, in individuals without any CMD component, there was no difference in lifespan associated with *FOXO3* genotype. Table 3 shows the association with just CHD, just diabetes, and just hypertension for each of the 8 *FOXO3* SNPs and the haplotype of each. Possession of the longevity-associated minor allele was associated with lower hazard ratio for mortality compared with major allele homozygotes in the CMD group when adjusted for age, and, in the full model, after adjustment for additional covariates that represent mortality risk factors.

**Table 1:** Age-adjusted baseline characteristics by CMD* and *FOXO* *rs2802292* genotype.

**Variable**	**With a CMD**			**Without a CMD**		
	***TT***	***GG*/*GT***	***P***	***TT***	***GG*/*GT***	***P***
n	1331	1181		570	502	
Age (years)	77.6 ± 4.5	78 ± 4.6	0.03	77.3 ± 4.4	78.1 ± 4.8	0.053
BMI (kg/m²)	23.7 ± 3.1	23.7 ± 3.0	0.62	22.8 ± 3	23 ± 3.0	0.37
Fasting plasma glucose (mg/dL)	116.8 ± 31.9	119.3 ± 35.2	0.060	101.3 ± 8.5	101.3 ± 8.6	1.00
Smoking (pack-years)	25.4 ± 33.4	28.5 ± 36.1	0.036	24.8 ± 33.4	23.9 ± 32.6	0.67
Alcohol intake (oz/mo)	19.6 ± 42.5	20.2 ± 44.3	0.76	16.2 ± 32.4	16.6 ± 33.4	0.85
Physical activity index	30.8 ± 4.8	30.8 ± 4.3	0.81	30.9 ± 4.3	31.2 ± 4.9	0.23
Depressive symptoms (%)	9.8	11.0	0.36	10.4	11.2	0.67
Stroke (%)	5.2	4.9	0.71	2.5	2.3	0.86
Cancer (%)	14.1	12.5	0.24	14.6	12.8	0.39
CHD (%)	31.7	26.7	0.01	–	–	–
Diabetes (%)	38.5	43.2	0.02	–	–	–
Hypertension (%)	75.9	77.0	0.50	–	–	–

Values shown are age-adjusted mean ± SD for indirect measures and proportion for direct measurements, except age.

*CMD: CHD or diabetes or hypertension

**Table 2:** Hazard ratios (HR) of *FOXO3* minor allele carriers vs. major allele homozygotes with total mortality by cardiometabolic disease (CMD)* status.

**Model**	***FOXO3* SNP**	**With a CMD (n=2512)**		**Without a CMD (n=1072)**	
		**HR (95% CI)**	***P***	**HR (95% CI)**	***P***
Age adjusted	*rs2802292*	0.88 (0.81-0.95)	0.0011	0.99 (0.88-1.12)	0.93
Age adjusted	*rs1935952*	0.90 (0.83-0.97)	0.0070	0.98 (0.87-1.12)	0.81
Age adjusted	*rs2253310*	0.88 (0.81-0.95)	0.0013	0.99 (0.88-1.12)	0.92
Age adjusted	*rs2764264*	0.90 (0.83-0.98)	0.012	0.97 (0.86-1.09)	0.61
Age adjusted	*rs2802288*	0.88 (0.81-0.95)	0.0012	0.99 (0.87-1.12)	0.85
Age adjusted	*rs3800230*	0.92 (0.84-1.00)	0.053	0.97 (0.84-1.11)	0.61
Age adjusted	*rs9398171*	0.90 (0.83-0.98)	0.012	0.98 (0.87-1.11)	0.77
Age adjusted	*rs12212067*	0.90 (0.81-1.00)	0.040	0.98 (0.84-1.14)	0.76
Age adjusted	*Haplotype*	0.86 (0.77-0.96)	0.0069	0.99 (0.84-1.17)	0.92
Covariate adjusted†	*rs2802292*	0.82 (0.75-0.89)	8.1E–06	1.03 (0.90-1.18)	0.63
Covariate adjusted	*rs1935952*	0.83 (0.76-0.91)	4.2E–05	1.06 (0.92-1.21)	0.44
Covariate adjusted	*rs2253310*	0.82 (0.75-0.90)	1.0E–05	1.04 (0.90-1.19)	0.61
Covariate adjusted	*rs2764264*	0.85 (0.78-0.93)	0.0002	1.03 (0.90-1.18)	0.65
Covariate adjusted	*rs2802288*	0.82 (0.75-0.89)	8.1E–06	1.03 (0.90-1.18)	0.70
Covariate adjusted	*rs3800230*	0.88 (0.80-0.97)	0.010	1.03 (0.89-1.20)	0.68
Covariate adjusted	*rs9398171*	0.84 (0.77-0.92)	0.0002	1.05 (0.92-1.21)	0.46
Covariate adjusted	*rs12212067*	0.88 (0.79-0.98)	0.023	1.05 (0.88-1.25)	0.59
Covariate adjusted	*Haplotype*^**¥**^	0.81 (0.72-0.91)	0.00031	1.07 (0.89-1.28)	0.49

*CMD: At least one of CHD, diabetes and hypertension.

^†^Covariates in Cox model: age, BMI, glucose, smoking pack-year, alcohol consumption (ounces per month), physical activity index (PAI), depressive symptoms, cancer and stroke.

^¥^Haplotype was obtained for 1,740 subjects with a CMD, and 742 subjects without a CMD.

Haplotype comprised minor allele frequencies of SNPs *rs2253310* (allele *C*), *rs2802288* (allele *A*), *rs2802292* (allele *G*), *rs2764264* (allele *C*), *rs9398171* (allele *C*), *rs12212067* (*G*), and *rs3800230* (allele *G*).

**Table 3:** Hazard ratios (HR) of *FOXO3* minor allele carriers vs. major allele homozygotes with total mortality by diabetes, CHD, and hypertension.

			**With a CMD**		**Without a CMD**	
**SNP**	**Cardiometabolic disease**	Model*	HR (95% CI) **	***P***	**HR (95% CI)**	***P***
*rs2802292*	Diabetes	1	0.84 (0.74-0.95)	0.0064	0.94 (0.87-1.01)	0.11
	(1010, 2525) ***	2	0.78 (0.68-0.89)	0.0003	0.93 (0.85-1.01)	0.08
	CHD	1	0.84 (0.73-0.97)	0.021	0.94 (0.88-1.02)	0.13
	(738, 2846)	2	0.79 (0.67-0.93)	0.0055	0.92 (0.85-1.00)	0.04
	Hypertension	1	0.89 (0.81-0.97)	0.0085	0.94 (0.85-1.04)	0.24
	(1919, 1665)	2	0.83 (0.75-0.92)	0.0003	0.94 (0.84-1.04)	0.22
*rs1935952*	Diabetes	1	0.84 (0.74-0.96)	0.0075	0.95 (0.88-1.03)	0.25
	(1010, 2521)	2	0.78 (0.68-0.90)	0.0005	0.95 (0.87-1.03)	0.22
	CHD	1	0.85 (0.73-0.98)	0.030	0.95 (0.88-1.03)	0.20
	(738, 2842)	2	0.81 (0.68-0.96)	0.015	0.93 (0.85-1.01)	0.08
	Hypertension	1	0.89 (0.82-0.98)	0.017	0.95 (0.86-1.05)	0.34
	(1917, 1663)	2	0.83 (0.75-0.92)	0.0002	0.97 (0.87-1.08)	0.60
*rs2253310*	Diabetes	1	0.84 (0.74-0.96)	0.0076	0.94 (0.87-1.01)	0.11
	(1010, 2523)	2	0.78 (0.69-0.90)	0.0004	0.93 (0.85-1.01)	0.08
	CHD	1	0.84 (0.73-0.97)	0.021	0.95 (0.88-1.02)	0.14
	(738, 2844)	2	0.79 (0.67-0.93)	0.0055	0.92 (0.85-1.00)	0.04
	Hypertension	1	0.89 (0.81-0.97)	0.0088	0.94 (0.86-1.04)	0.25
	(1918, 1664)	2	0.83 (0.75-0.92)	0.0003	0.94 (0.84-1.04)	0.24
*rs2764264*	Diabetes	1	0.85 (0.75-0.96)	0.0097	0.95 (0.88-1.03)	0.20
	(1009, 2521)	2	0.80 (0.70-0.92)	0.0014	0.95 (0.87-1.04)	0.25
	CHD	1	0.85 (0.73-0.99)	0.034	0.95 (0.89-1.03)	0.21
	(736, 2843)	2	0.81 (0.69-0.96)	0.015	0.94 (0.86-1.02)	0.13
	Hypertension	1	0.91 (0.83-1.00)	0.041	0.94 (0.85-1.03)	0.20
	(1915, 1664)	2	0.86 (0.78-0.95)	0.0028	0.95 (0.85-1.06)	0.38
*rs2802288*	Diabetes	1	0.84 (0.74-0.95)	0.0062	0.93 (0.86-1.01)	0.10
	(1009, 2516)	2	0.78 (0.68-0.89)	0.0002	0.92 (0.85-1.01)	0.07
	CHD	1	0.84 (0.72-0.97)	0.0182	0.94 (0.88-1.02)	0.13
	(736, 2838)	2	0.79 (0.67-0.93)	0.0047	0.92 (0.84-0.99)	0.03
	Hypertension	1	0.89 (0.81-0.97)	0.0084	0.94 (0.85-1.04)	0.22
	(1915, 1659)	2	0.83 (0.75-0.92)	0.0003	0.93 (0.84-1.04)	0.19
*rs3800230*	Diabetes	1	0.89 (0.77-1.02)	0.1038	0.96 (0.88-1.05)	0.38
	(998, 2501)	2	0.88 (0.76-1.02)	0.095	0.93 (0.85-1.03)	0.17
	CHD	1	0.96 (0.81-1.14)	0.68	0.94 (0.86-1.02)	0.12
	(731, 2817)	2	0.92 (0.76-1.11)	0.3832	0.93 (0.85-1.01)	0.10
	Hypertension	1	0.92 (0.83-1.02)	0.099	0.94 (0.85-1.05)	0.30
	(1901, 1647)	2	0.89 (0.79-0.99)	0.033	0.95 (0.84-1.07)	0.38
*rs9398171*	Diabetes	1	0.86 (0.76-0.97)	0.015	0.95 (0.88-1.03)	0.21
	(1002, 2508)	2	0.81 (0.70-0.92)	0.0017	0.95 (0.87-1.04)	0.24
	CHD	1	0.85 (0.73-0.99)	0.032	0.96 (0.89-1.03)	0.26
	(732, 2827)	2	0.81 (0.68-0.96)	0.013	0.94 (0.87-1.02)	0.14
	Hypertension	1	0.91 (0.83-0.99)	0.033	0.95 (0.86-1.05)	0.30
	(1905, 1654)	2	0.85 (0.77-0.94)	0.0019	0.96 (0.86-1.07)	0.50
*rs12212067*	Diabetes	1	0.92 (0.78-1.08)	0.31	0.95 (0.86-1.05)	0.28
	(1005, 2502)	2	0.88 (0.74-1.05)	0.15	0.94 (0.84-1.05)	0.27
	CHD	1	0.90 (0.74-1.10)	0.30	0.94 (0.86-1.03)	0.19
	(732, 2824)	2	0.90 (0.73-1.12)	0.36	0.93 (0.84-1.03)	0.17
	Hypertension	1	0.90 (0.80-1.00)	0.056	0.96 (0.85-1.09)	0.51
	(1903, 1653)	2	0.88 (0.78-0.99)	0.037	0.96 (0.84-1.11)	0.60
Haplotype****	Diabetes	1	0.83 (0.70-0.99)	0.034	0.94 (0.85-1.05)	0.26
	(661, 1793)	2	0.77 (0.64-0.93)	0.0071	0.93 (0.82-1.04)	0.19
	CHD	1	0.85 (0.69-1.05)	0.12	0.93 (0.84-1.03)	0.15
	(526, 1956)	2	0.83 (0.66-1.04)	0.10	0.90 (0.81-1.01)	0.07
	Hypertension	1	0.87 (0.77-0.98)	0.022	0.94 (0.81-1.09)	0.43
	(1331, 1151)	2	0.82 (0.72-0.93)	0.0003	0.93 (0.84-1.04)	0.23

* 1: Age-adjusted; 2: Covariate-adjusted, Covariates in Cox model: age, BMI, glucose, smoking (pack-year), alcohol intake (oz/mo), physical activity index, depression, cancer, and stroke.

** HR (95% CI) of *FOXO3* minor allele carrier vs. major allele homozygotes on mortality estimated from the Cox regression model.

*** Number of subjects with a CMD, without a CMD.

**** Haplotype comprised minor allele frequencies of SNPs *rs2253310* (C)*, rs2802288* (*A*)*, rs2802292* (*G*)*, rs2764264* (*C*)*, rs9398171* (*C*)*, rs12212067* (*G*)*,* and *rs3800230* (*G*)*.*

## DISCUSSION

Here we show for the first time that longevity-associated genetic variants of *FOXO3* are associated with much lower mortality in elderly individuals who have one or more of the common aging-related conditions consisting of diabetes, CHD and hypertension. In contrast, no reduction in mortality was observed in men with a CMD who did not have the longevity genotype. In addition, surprisingly, in men with CMD and the *FOXO3* longevity genotype, mortality was reduced to normal (i.e., to that of men without a CMD). We propose that longevity-associated *FOXO3* variants confer resilience against the adverse medical conditions that comprise CMD.

FoxO3 serves as a core regulator of cellular homeostasis, stress response, and longevity through its ability to modulate a variety of stress responses during nutrient shortage, oxidative stress, hypoxia, heat shock, and DNA damage [14–17]. By reducing oxidative damage responsible for aging, FoxO3-mediated responses to stress are pivotal to health-span and lifespan [18]. Depending on the stress stimulus and subcellular context, once activated, FoxO3 can induce specific sets of nuclear genes, including cell cycle inhibitors, pro-apoptotic genes, scavengers of reactive oxygen species, autophagy effectors, gluconeogenic enzymes, and others [17]. On the other hand, under glucose restriction, FoxO3 translocates to mitochondria to stimulate transcription of oxidative phosphorylation genes, thus restoring cellular ATP levels [17]. FoxO3 target genes and the pathways that their gene products serve are diverse and sometimes antagonistic, meaning FoxO3 is an adaptable player in the dynamic homeostasis of normal and stressed cells [17].

In unstimulated lymphoblast cell lines, *FOXO3* mRNA expression is significantly higher in *rs2802292* *GT* vs. *TT* cells, and in response to H_2_O_2_-mediated stress, expression increases for each genotype (*P* < 0.0001), reaching a 3-fold higher level in *GT* than *TT* cells [8]. In this study fluorescence in situ hybridization showed that the nuclear position of *FOXO3* changed with regards to its neighbours over a >7 Mb region following stress induction, bringing distant genes into proximity with *FOXO3.* These data indicated that the longevity haplotype is better “primed” for stress response than the common haplotype [8]. If there is no cellular stress, then *FOXO3* appears to play only a small part in cell resilience.

The coronary artery and aortic arch are prone to atherosclerosis and thus CHD. FoxO3 is a central protective factor in safeguarding primate vascular homeostasis – it serves as a master regulator and key driver of aortic and coronary vascular endothelial aging [19]. FoxO3 is the top regulatory factor affecting primate vascular aging, having the highest number of target genes [19]. In various tissues, dysregulation of the developmental program is increasingly regarded as pivotal to cellular aging [20, 21]. A key player in this process is the developmental regulator FOXA2, which becomes upregulated in aging vessels. Crucially, in human aortic endothelial cells, FOXA2 binding to the *FOXO3* promoter suppresses *FOXO3* expression and compromises cell proliferation, the effect being reversed by siRNA-mediated knockdown of *FOXA2* expression [19]. Interestingly the most 5’ SNP in our *FOXO3* longevity haplotype was *rs768023*, located in the promoter region, and which we noted previously is the binding site for both FOXA2 and HDAC2 [8]. HDAC2 also deacetylates FoxO3 thus activating it [22]. For the *rs768023* minor allele, binding of FOXA2 and HDAC is abolished, which would increase *FOXO3* expression. The SNP immediately 3’ of *rs768023* in the 14-SNP haplotype block, but not included in the present study, is *rs1536057*, the minor (*A*) allele of which abolishes NRF1 and E2F sites and creates a POU5F1 site. The inhibition of *FOXO3* expression by E2F [23] would thereby be lost, so causing an elevation in *FOXO3* expression. NRF1 and POU5FI are involved in cell proliferation and stem cell maintenance, while E2F is involved in DNA damage response.

The full complement of transcription factors that exhibit allele-dependent binding to SNPs in the 14-SNP *FOXO3* haplotype block are shown in Supplementary Table 2. Of these, HDAC1, NRF1, POU5F1, TFCP2L1, MYOG, NKX3-1, MITF, MEF2A, MZF1, NR2F1, PRDM1, FOXP1, MZF1, and ONECUT1 are involved in cell proliferation/stem cell maintenance, whereas HNF3, FOXA2, HNF4A, and ONECUT1 (HNF6) are involved in diabetes/energy response, and E2F1 is involved in DNA damage repair.

We have shown previously that the *G* allele of *FOXO3* SNP *rs2802292* protects against risk of death from CHD [11]. *G* allele carriers had lower plasma TNF-α than non-carriers, suggesting an ability to reduce inflammation as a potential mediating factor for reduction of CHD mortality risk [24]. At the cell and molecular level, cardiovascular benefits of FoxO3 involve stimulation of stress resistance by up-regulation of mitochondrial superoxide dismutase, peroxisomal catalase, and peroxiredoxin expression [25], as well as the promotion of multiple beneficial vascular functions, and reversal of cellular aging through pro-proliferative effects that involve downregulation of CSRP1, which recruits sirtuin-1 to create a suppressive chromatin environment [26]. Longevity-associated genetic variants of *FOXO3* are also associated with lower blood pressure and reduced hypertension prevalence [27]. The association of *FOXO3* SNPs with *self-rated* health in individuals aged 75–87 is influenced solely by cardiovascular disease [28].

Various types of cellular stress induce the recruitment of the evolutionarily conserved transcription factor HSF1 to binding sites involved in controlling gene expression [29]. The fact that only in *G* allele carriers of *FOXO3* SNP *rs2802292* possess the HSF1 binding site [30] explains, at least in part, the association we found between this allele and resistance to mortality in CMD subjects, presumably mediated by HSF1-induced expression of *FOXO3*. Binding of HSF1 to its target site in *rs2802292* leads to formation of a complex resulting in a promoter-enhancer interaction involving the upstream promoter region and the *rs2802292* region of intron 2 [30]. Stress resistance and elevated *FOXO3* transcription were seen following application of the potent oxidative stress inducer H_2_O_2_ in human dermal fibroblasts having the *GG* genotype, but not in those with the *TT* genotype, thus confirming the mechanism [30]. Unlike cells with the *TT* genotype, *GG* cells also exhibited higher expression of FoxO3 target genes *SOD2*, *CAT*, *GADD45A*, *CCND1*, *RBL2*, *BCL2L11* and *BCL6* in a HSF1-dependent manner [30]. Furthermore, *GG* cells displayed a significantly better DNA repair response. The *FOXO3* SNP *rs2802292* region also contains transcription factor response elements for SP1, GATA1 and ESR1 [30].

Our finding of 13% higher prevalence of diabetes at baseline in *FOXO3 rs2800292* *G*-allele carriers is likely due to attrition of non-protective *TT* genotypes at earlier ages owing to mortality from cardiovascular disease (e.g., CHD, stroke) and other complications of diabetes, thereby enriching *G*-allele frequency in the cohort. In a twin study, the *FOXO3 rs2800292* *G* allele was associated with more favourable insulin sensitivity and increased *FOXO3* expression [31]. The protection against mortality afforded by this mechanism would explain our finding that diabetes alone was associated with longer lifespan in *G*-allele carriers compared with *TT* subjects. As we have reported previously, the *FOXO3* longevity genotype involves a haplotype that includes at least 14 SNPs that work in concert [8].

We reported previously that the *FOXO3 rs2800292* *G* allele was associated with reduced risk of hypertension in women, but not men, from a cohort of Japanese individuals living in Japan [32]. Our finding in the present study that *G*-allele carriers have lower mortality could, as shown here for individuals with diabetes, be due to a higher mortality rate for those with the *TT* genotype, in this instance from hypertension-related cardiovascular events, similar to what has been demonstrated by one of us for *ACE* [33].

In conclusion, we have found for the first time that remaining lifespan of elderly men with longevity-associated alleles of *FOXO3* who have one or more chronic conditions of aging, specifically diabetes, and/or hypertension, and/or CHD (collectively, CMD), live as long as elderly men who lack any of these life-threatening conditions. Thus, favorable *FOXO3* genotype can abrogate the increased risk for mortality in men with prevalent CMD. Since *FOXO3* genotype has no effect on lifespan of men without a *CMD*, the well-established understanding of *FOXO3* as being a longevity gene is because of an effect on elderly individuals who are at higher risk because they have a CMD.

## MATERIALS AND METHODS

### Study cohort

Participants were American men of Japanese ancestry living on the island of Oahu, Hawaii. They were recruited in 1965–1968 from World War II Selective Service records for the Kuakini Honolulu Heart Program (KHHP) [34], which continued from 1991 onwards as the Kuakini and Honolulu-Asia Aging Study (KHAAS) [4, 34–36]. The analysis was conducted as part of the Kuakini Hawaii Lifespan Study and the Kuakini Hawaii Healthspan Study, an embedded cohort study of healthy aging drawn from the original KHHP-KHAAS population. Subjects had parents who were both from a limited geographic area of Japan, mostly the western, central and southern regions [34, 37]. Subjects were recruited at the same time and place (Oahu), meaning there was no apparent reason why genetic background should be substantially different. The KHHP cohort is quite robust for phenotype-genotype associations, since the data collection was exceptionally accurate and involved cross validation utilizing an expert Morbidity and Mortality Committee. The Hawaii Japanese population is from Japan, with little outbreeding and, based on the authors’ unpublished data, exhibits a smaller degree of genetic diversity than the overall population of Japan.

All participants in the current study were interviewed at Examination 4 of the KHHP (1991–1993). Archived phenotypic data and blood samples from Examination 4 of the KHHP (1991–1993), which coincided with the commencement of the KHAAS, were used as the baseline examination for our study. The KHAAS was begun as an expansion of the KHHP for the study of neurodegenerative diseases, cognitive function, and other aging phenotypes in elderly persons. From 1991–1993, all of the survivors of the KHHP cohort, ranging in age from 71–93 years (mean age: 77.9 ± 4.7 years), were invited to the 4^th^ examination. Response rate was 80% of survivors (including clinic, home, and nursing home visits; n = 3,741).

The study involved 3,584 of 3,741 men aged 71 to 93 (mean age 77.9 ± 4.7 SD years) for whom we had banked DNA so making them eligible for inclusion. Of the 3,584 men, 3,548 had died (mean age at death 89.0±6.2 SD years; range 72–108 years) and 36 were still alive (mean age 101.6 ± 1.9 SD years; range 100–108 years) at the end of the follow-up period, 31 December 2019.

The KHHP was a longitudinal observation study. Subjects from the KHHP/KHAAS population [4] had been followed with regular examinations and blood work until 2015, or death up to the end of 2019.

Information on the prevalence of CHD, stroke, and cancer was identified by the KHHP surveillance system (review of hospital records by an expert panel or matching to Tumor Registry for Cancer). Hypertension was defined as systolic/diastolic blood pressure ≥160/95 mmHg or on anti-hypertensive medication at baseline. Diabetes was defined by fasting serum glucose ≥126 mg/dL or 2-hour post-load glucose ≥200 mg/dL or taking insulin and/or oral hypoglycemic medications at baseline.

Procedures performed were in accord with institutional guidelines and were approved by the Institutional Review Board of Kuakini Medical Center. Written informed consent was obtained from all study participants or from family representatives, if participants could not provide consent.

### Genotyping

Leucocyte DNA obtained from participants was used for genotyping. Recruitment, demographic characteristics, and DNA extraction were as described previously [4, 38]. In the HHP cohort, the *FOXO3* longevity haplotype was defined by 14 SNPs (in order of location 5’ to 3’ with minor [longevity-associated] allele shown in brackets): *rs768023* (*G* allele), *rs1536057* (*T*), *rs2253310* (C), *rs2802288* (*A*), *rs2802292 (G),* *rs2764264* (*C*), *rs12202234* (*G*), *rs17069665* (*G*), *rs12213895 (A),* *rs12212067* (*G*), *rs9398171* (*C*), *rs73763159* (*T*), *rs3800230* (*G*), and *rs1935952* (*G*)) [8]. Longitudinal genotype data were obtained for 8 of these SNPs. All 14 affect transcription factor binding sites. Specifically, the minor allele of *rs2802292* creates a HSF1 binding site in intron 2 of *FOXO3* [30], *rs1935952* disrupts an MZF1 binding site, *rs2253310* minor allele creates a TFCP2L1 binding site, *rs2764264* disrupts a NKX3 binding site, *rs2802288* creates a MYF binding site, *rs3800230* disrupts a FoxP1 binding site, *rs9398171* *creates* NR2F1 and HNF4 site and abolishes a HNF6 site, and *rs12212067* creates a MZF1 site [8]. The SNP *rs768023* (not included in the present longitudinal study) abolishes both a FOXA2 and a HDAC2 recognition site [8].

Genotyping was performed by allelic discrimination assays using TaqMan^®^ (Applied Biosystems, Inc.) and a Life Technologies QuantStudio 12K Flex OpenArray system. Although 88% of participants were born in Hawaii, there is a theoretical possibility of confounding of case vs. control status for allele frequencies due to geographic origin. Therefore, for certain analyses, cases and controls were stratified by parental prefecture of origin using conditional logistic regression models.

### Statistical analyses

General linear models were used to compare age-adjusted indirect measurements between groups, and logistic models were used to compare the age-adjusted direct measurements. Cox proportional models were used to assess the association of *FOXO3* minor-allele carriers with mortality stratified by disease status, such as by CHD, by diabetes, by hypertension, and by chronic conditions defined by presence of any of CHD, diabetes, or hypertension. The Cox proportional hazard assumption was tested for each Cox model. The effect of interaction of disease with *FOXO* genotype on mortality was tested in the Cox model. All statistical analyses were performed using the Statistical Analysis System version 9.4 [39]. Figures were generated using the STATA 12 Graphics [40].

## Supplementary Material

Supplementary Methods

Supplementary Figures

Supplementary Tables

## References

[1] Morris BJ, Willcox BJ, Donlon TA. Genetic and epigenetic regulation of human aging and longevity. Biochim Biophys Acta Mol Basis Dis. 2019; 1865:1718–44. 10.1016/j.bbadis.2018.08.039 31109447 PMC7295568

[2] Brooks-Wilson AR. Genetics of healthy aging and longevity. Hum Genet. 2013; 132:1323–38. 10.1007/s00439-013-1342-z 23925498 PMC3898394

[3] Berg NV, Rodríguez-Girondo M, de Craen AJ, Houwing-Duistermaat JJ, Beekman M, Slagboom PE. Longevity around the turn of the 20^th^ century: life-long sustained survival advantage for parents of today’s nonagenarians. J Gerontol A Biol Sci Med Sci. 2018; 73:1295–302. 10.1093/gerona/gly049 29596573 PMC6132126

[4] Willcox BJ, Donlon TA, He Q, Chen R, Grove JS, Yano K, Masaki KH, Willcox DC, Rodriguez B, Curb JD. FOXO3A genotype is strongly associated with human longevity. Proc Natl Acad Sci USA. 2008; 105:13987–92. 10.1073/pnas.0801030105 18765803 PMC2544566

[5] Broer L, Buchman AS, Deelen J, Evans DS, Faul JD, Lunetta KL, Sebastiani P, Smith JA, Smith AV, Tanaka T, Yu L, Arnold AM, Aspelund T, et al. GWAS of longevity in CHARGE consortium confirms APOE and FOXO3 candidacy. J Gerontol A Biol Sci Med Sci. 2015; 70:110–18. 10.1093/gerona/glu166 25199915 PMC4296168

[6] Morris BJ, Willcox DC, Donlon TA, Willcox BJ. FOXO3: a major gene for human longevity—a mini-review. Gerontology. 2015; 61:515–25. 10.1159/000375235 25832544 PMC5403515

[7] Deelen J, Evans DS, Arking DE, Tesi N, Nygaard M, Liu X, Wojczynski MK, Biggs ML, van der Spek A, Atzmon G, Ware EB, Sarnowski C, Smith AV, et al. A meta-analysis of genome-wide association studies identifies multiple longevity genes. Nat Commun. 2019; 10:3669. 10.1038/s41467-019-11558-2 31413261 PMC6694136

[8] Donlon TA, Morris BJ, Chen R, Masaki KH, Allsopp RC, Willcox DC, Elliott A, Willcox BJ. FOXO3 longevity interactome on chromosome 6. Aging Cell. 2017; 16:1016–25. 10.1111/acel.12625 28722347 PMC5595686

[9] Eijkelenboom A, Mokry M, de Wit E, Smits LM, Polderman PE, van Triest MH, van Boxtel R, Schulze A, de Laat W, Cuppen E, Burgering BM. Genome-wide analysis of FOXO3 mediated transcription regulation through RNA polymerase II profiling. Mol Syst Biol. 2013; 9:638. 10.1038/msb.2012.74 23340844 PMC3564262

[10] Donlon TA, Willcox BJ, Morris BJ. FOXO3 cell resilience gene neighborhood. Aging (Albany NY). 2017; 9:2467–68. 10.18632/aging.101349 29242406 PMC5764384

[11] Willcox BJ, Tranah GJ, Chen R, Morris BJ, Masaki KH, He Q, Willcox DC, Allsopp RC, Moisyadi S, Poon LW, Rodriguez B, Newman AB, Harris TB, et al. The FoxO3 gene and cause-specific mortality. Aging Cell. 2016; 15:617–24. 10.1111/acel.12452 27071935 PMC4933667

[12] Pawlikowska L, Hu D, Huntsman S, Sung A, Chu C, Chen J, Joyner AH, Schork NJ, Hsueh WC, Reiner AP, Psaty BM, Atzmon G, Barzilai N, et al, and Study of Osteoporotic Fractures. Association of common genetic variation in the insulin/IGF1 signaling pathway with human longevity. Aging Cell. 2009; 8:460–72. 10.1111/j.1474-9726.2009.00493.x 19489743 PMC3652804

[13] Kuningas M, Mägi R, Westendorp RG, Slagboom PE, Remm M, van Heemst D. Haplotypes in the human Foxo1a and Foxo3a genes; impact on disease and mortality at old age. Eur J Hum Genet. 2007; 15:294–301. 10.1038/sj.ejhg.5201766 17245409

[14] Kops GJ, Dansen TB, Polderman PE, Saarloos I, Wirtz KW, Coffer PJ, Huang TT, Bos JL, Medema RH, Burgering BM. Forkhead transcription factor FOXO3a protects quiescent cells from oxidative stress. Nature. 2002; 419:316–21. 10.1038/nature01036 12239572

[15] Sengupta A, Molkentin JD, Paik JH, DePinho RA, Yutzey KE. FoxO transcription factors promote cardiomyocyte survival upon induction of oxidative stress. J Biol Chem. 2011; 286:7468–78. 10.1074/jbc.M110.179242 21159781 PMC3045002

[16] Webb AE, Brunet A. FOXO transcription factors: key regulators of cellular quality control. Trends Biochem Sci. 2014; 39:159–69. 10.1016/j.tibs.2014.02.003 24630600 PMC4021867

[17] Fasano C, Disciglio V, Bertora S, Lepore Signorile M, Simone C. FOXO3a from the nucleus to the mitochondria: a round trip in cellular stress response. Cells. 2019; 8:1110. 10.3390/cells8091110 31546924 PMC6769815

[18] Gurkar AU, Robinson AR, Cui Y, Li X, Allani SK, Webster A, Muravia M, Fallahi M, Weissbach H, Robbins PD, Wang Y, Kelley EE, Croix CM, et al. Dysregulation of DAF-16/FOXO3A-mediated stress responses accelerates oxidative DNA damage induced aging. Redox Biol. 2018; 18:191–99. 10.1016/j.redox.2018.06.005 30031267 PMC6076207

[19] Zhang W, Zhang S, Yan P, Ren J, Song M, Li J, Lei J, Pan H, Wang S, Ma X, Ma S, Li H, Sun F, et al. A single-cell transcriptomic landscape of primate arterial aging. Nat Commun. 2020; 11:2202. 10.1038/s41467-020-15997-0 32371953 PMC7200799

[20] Ermolaeva M, Neri F, Ori A, Rudolph KL. Cellular and epigenetic drivers of stem cell ageing. Nat Rev Mol Cell Biol. 2018; 19:594–610. 10.1038/s41580-018-0020-3 29858605

[21] Whitton H, Singh LN, Patrick MA, Price AJ, Osorio FG, López-Otín C, Bochkis IM. Changes at the nuclear lamina alter binding of pioneer factor Foxa2 in aged liver. Aging Cell. 2018; 17:e12742. 10.1111/acel.12742 29484800 PMC5946061

[22] Beharry AW, Sandesara PB, Roberts BM, Ferreira LF, Senf SM, Judge AR. HDAC1 activates FoxO and is both sufficient and required for skeletal muscle atrophy. J Cell Sci. 2014; 127:1441–53. 10.1242/jcs.136390 24463822 PMC3970557

[23] Xie Q, Peng S, Tao L, Ruan H, Yang Y, Li TM, Adams U, Meng S, Bi X, Dong MQ, Yuan Z. E2F transcription factor 1 regulates cellular and organismal senescence by inhibiting forkhead box O transcription factors. J Biol Chem. 2014; 289:34205–13. 10.1074/jbc.M114.587170 25344604 PMC4256352

[24] Willcox BJ, Morris BJ, Tranah GJ, Chen R, Masaki KH, He Q, Willcox DC, Allsopp RC, Moisyadi S, Gerschenson M, Davy PM, Poon LW, Rodriguez B, et al. Longevity-associated FOXO3 genotype and its impact on coronary artery disease mortality in Japanese, whites, and blacks: a prospective study of three American populations. J Gerontol A Biol Sci Med Sci. 2017; 72:724–28. 10.1093/gerona/glw196 27694344 PMC5964743

[25] Chiribau CB, Cheng L, Cucoranu IC, Yu YS, Clempus RE, Sorescu D. FOXO3A regulates peroxiredoxin III expression in human cardiac fibroblasts. J Biol Chem. 2008; 283:8211–17. 10.1074/jbc.M710610200 18195003 PMC2276380

[26] Yan P, Li Q, Wang L, Lu P, Suzuki K, Liu Z, Lei J, Li W, He X, Wang S, Ding J, Chan P, Zhang W, et al. FOXO3-engineered human ESC-derived vascular cells promote vascular protection and regeneration. Cell Stem Cell. 2019; 24:447–61.e8. 10.1016/j.stem.2018.12.002 30661960

[27] International Consortium for Blood Pressure Genome-Wide Association Studies, Ehret GB, Munroe PB, Rice KM, Bochud M, Johnson AD, Chasman DI, Smith AV, Tobin MD, Verwoert GC, Hwang SJ, Pihur V, Vollenweider P, O'Reilly PF, et al. Genetic variants in novel pathways influence blood pressure and cardiovascular disease risk. Nature. 2011; 478:103–9. 10.1038/nature10405 21909115 PMC3340926

[28] Zettergren A, Kern S, Rydén L, Östling S, Blennow K, Zetterberg H, Falk H, Skoog I. Genetic variation in FOXO3 is associated with self-rated health in a population-based sample of older individuals. J Gerontol A Biol Sci Med Sci. 2018; 73:1453–58. 10.1093/gerona/gly021 29415201 PMC6175024

[29] Vihervaara A, Sistonen L. HSF1 at a glance. J Cell Sci. 2014; 127:261–66. 10.1242/jcs.132605 24421309

[30] Grossi V, Forte G, Sanese P, Peserico A, Tezil T, Lepore Signorile M, Fasano C, Lovaglio R, Bagnulo R, Loconte DC, Susca FC, Resta N, Simone C. The longevity SNP rs2802292 uncovered: HSF1 activates stress-dependent expression of FOXO3 through an intronic enhancer. Nucleic Acids Res. 2018; 46:5587–600. 10.1093/nar/gky331 29733381 PMC6009585

[31] Banasik K, Ribel-Madsen R, Gjesing AP, Wegner L, Andersson A, Poulsen P, Borglykke A, Witte DR, Pedersen O, Hansen T, Vaag A. The FOXO3A rs2802292 G-allele associates with improved peripheral and hepatic insulin sensitivity and increased skeletal muscle-FOXO3A mRNA expression in twins. J Clin Endocrinol Metab. 2011; 96:E119–24. 10.1210/jc.2010-0881 20881262

[32] Morris BJ, Chen R, Donlon TA, Evans DS, Tranah GJ, Parimi N, Ehret GB, Newton-Cheh C, Seto T, Willcox DC, Masaki KH, Kamide K, Ryuno H, et al. Association analysis of FOXO3 longevity variants with blood pressure and essential hypertension. Am J Hypertens. 2016; 29:1292–300. 10.1093/ajh/hpv171 26476085 PMC5055732

[33] Morris BJ, Zee RY, Schrader AP. Different frequencies of angiotensin-converting enzyme genotypes in older hypertensive individuals. J Clin Invest. 1994; 94:1085–89. 10.1172/JCI117423 8083349 PMC295169

[34] Worth RM, Kagan A. Ascertainment of men of Japanese ancestry in hawaii through world war II selective service registration. J Chronic Dis. 1970; 23:389–97. 10.1016/0021-9681(70)90022-6 5492969

[35] Kagan A, Harris BR, Winkelstein W Jr, Johnson KG, Kato H, Syme SL, Rhoads GG, Gay ML, Nichaman MZ, Hamilton HB, Tillotson J. Epidemiologic studies of coronary heart disease and stroke in Japanese men living in Japan, Hawaii and California: demographic, physical, dietary and biochemical characteristics. J Chronic Dis. 1974; 27:345–64. 10.1016/0021-9681(74)90014-9 4436426

[36] Yano K, Reed DM, McGee DL. Ten-year incidence of coronary heart disease in the Honolulu Heart Program. Relationship to biologic and lifestyle characteristics. Am J Epidemiol. 1984; 119:653–66. 10.1093/oxfordjournals.aje.a113787 6720665

[37] Kagan A, Ed. (1996) The Honolulu Heart Program: An Epidemiological Study of Coronary Heart Disease and Stroke. Amsterdam, The Netherlands: Harwood Academic Publishers.

[38] Donlon TA, Curb JD, He Q, Grove JS, Masaki KH, Rodriguez B, Elliott A, Willcox DC, Willcox BJ. FOXO3 gene variants and human aging: coding variants may not be key players. J Gerontol A Biol Sci Med Sci. 2012; 67:1132–39. 10.1093/gerona/gls067 22459618 PMC3668389

[39] Statistical Analysis System (SAS) version 9.4. SAS Institute, Cary, NC, USA.. https://libguides.library.kent.edu/statconsulting/SAS.

[40] StataCorp LP. Stata Statistical Software: Release 12. College Station, TX. 2011 https://www.coursehero.com/file/16695740/stata/

