# Supplementary material for: FOXO3 longevity genotype mitigates the increased mortality risk in men with a cardiometabolic disease
Source: Aging (Albany NY). 2020 Dec 1;12(23):23509–24. doi: 10.18632/aging.202175 (PMC7762472; doi:10.18632/aging.202175)
Supplement: Supplementary Methods [file aging-12-202175-s001.pdf]

Supplementary Methods

Chen R, Morris BJ, Donlon TA, Maskai KH, Willcox DC, Davy PMC, Allsopp RC, Willcox BJ. ***FOXO3* longevity genotype mitigates the increased mortality risk in men with a cardiometabolic disease.**
