# Supplementary material for: FOXO3 longevity genotype mitigates the increased mortality risk in men with a cardiometabolic disease
Source: Aging (Albany NY). 2020 Dec 1;12(23):23509–24. doi: 10.18632/aging.202175 (PMC7762472; doi:10.18632/aging.202175)
Supplement: Supplementary Figures [file aging-12-202175-s002.pdf]

SUPPLEMENTARY FIGURES

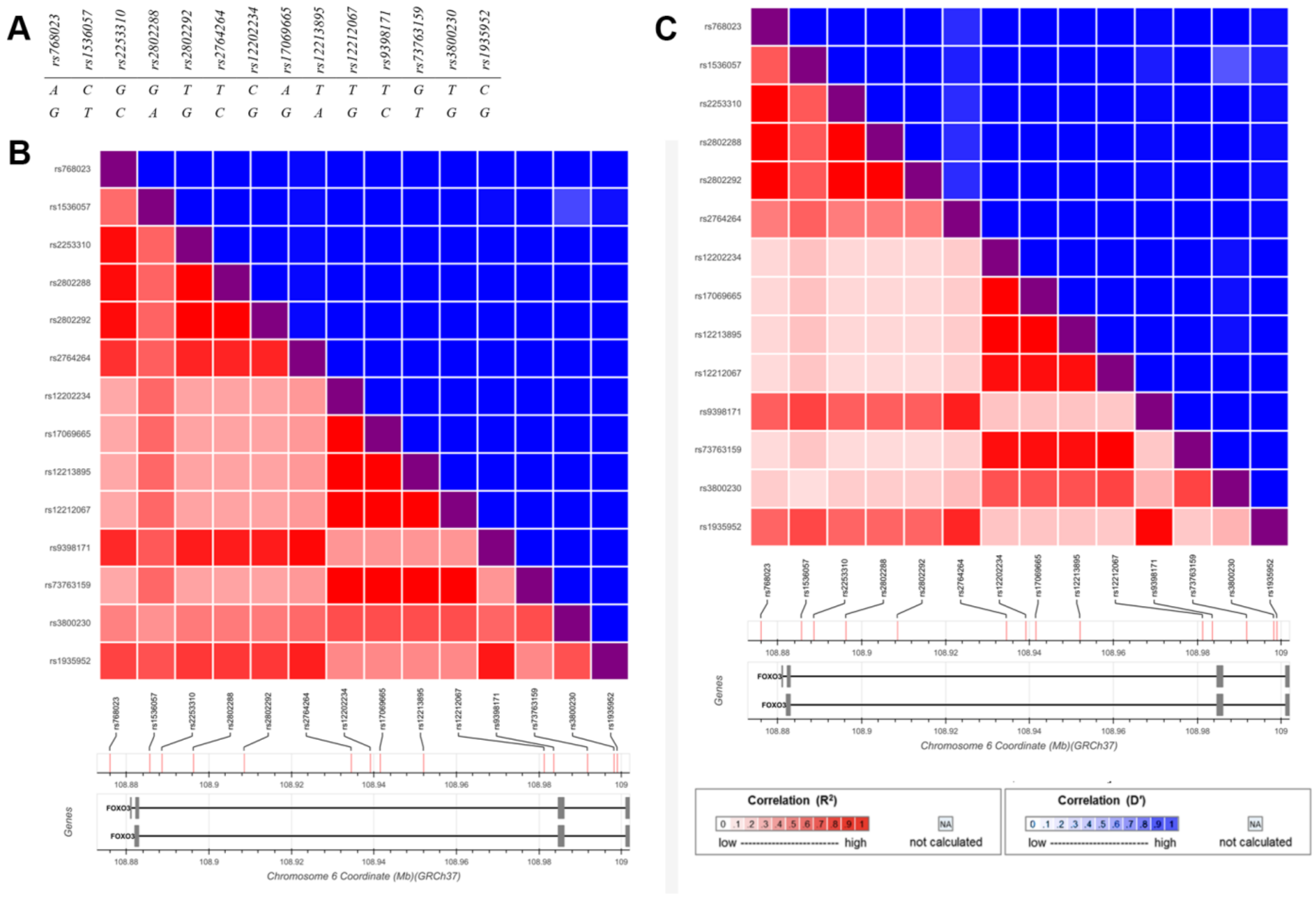

Supplementary Figure 1. Linkage disequilibrium and haplotypes of 14 *FOXO3* longevity-associated SNPs. (**A**) The 14 *FOXO3* longevity-associated SNPs (and alleles) that comprise the full haplotype. (**B**) Linkage disequilibrium in *FOXO3* and SNP locations – Japanese (JPN). (**C**) Linkage disequilibrium in *FOXO3* and SNP locations – Caucasian (CEU). LD matrix plot showing the 14 *FOXO3* SNPs using the program LDLink (https://ldlink.nci.nih.gov). In the Japanese population, shown in part (**B**) of Supplementary Figure 1, the data are from “JPN” population data from Phase 3 (Version 5) of the 1000 Genomes Project (https://www.internationalgenome.org). In the Caucasian population the values are from the “CEU” data. Note that the JPN data have a higher overall level of linkage disequilibrium (i.e., red intensities). Red squares denote blocks that have a Hedrick’s multiallelic R^2 = 1, whereas blue squares denote blocks that have a R^2 value < 1 [Hedrick PW. Gametic disequilibrium measures: Proceed with caution. *Genetics.* 1987; 117:331-342]. Blue blocks denote *D’* values.

A

RS Number	Position (GRCh37)	Allele Frequencies	Haplotypes					
rs768023	chr6:108876002	A=0.755, G=0.245	A	G	G	G	G	G
rs1536057	chr6:108885623	C=0.841, T=0.159	C	T	T	C	C	C
rs2253310	chr6:108888593	G=0.764, C=0.236	G	C	C	C	C	C
rs2802288	chr6:108896215	G=0.764, A=0.236	G	A	A	A	A	A
rs2802292	chr6:108908518	T=0.764, G=0.236	T	G	G	G	G	G
rs2764264	chr6:108934461	T=0.784, C=0.216	T	C	C	C	T	C
rs12202234	chr6:108939083	C=0.899, G=0.101	C	G	C	C	C	C
rs17069665	chr6:108941468	A=0.899, G=0.101	A	G	A	A	A	A
rs12213895	chr6:108951968	T=0.899, A=0.101	T	A	T	T	T	T
rs12212067	chr6:108981196	T=0.899, G=0.101	T	G	T	T	T	T
rs9398171	chr6:108983527	T=0.788, C=0.211	T	C	C	C	T	C
rs73763159	chr6:108991686	G=0.899, T=0.101	G	T	G	G	G	G
rs3800230	chr6:108998128	T=0.861, G=0.139	T	G	T	G	T	T
rs1935952	chr6:108998905	C=0.808, G=0.192	C	G	G	G	C	C
		Haplotype Count	156	21	9	7	4	3
		Haplotype Frequency	0.75	0.101	0.0433	0.0337	0.0192	0.0144

B

RS Number	Position (GRCh37)	Allele Frequencies	Haplotypes						
rs768023	chr6:108876002	A=0.646, G=0.353	A	G	G	G	A	G	G
rs1536057	chr6:108885623	C=0.737, T=0.263	C	T	T	C	C	C	T
rs2253310	chr6:108888593	G=0.646, C=0.353	G	C	C	C	G	C	C
rs2802288	chr6:108896215	G=0.646, A=0.353	G	A	A	A	G	A	A
rs2802292	chr6:108908518	T=0.646, G=0.353	T	G	G	G	T	G	G
rs2764264	chr6:108934461	T=0.702, C=0.298	T	C	C	T	C	C	T
rs12202234	chr6:108939083	C=0.919, G=0.081	C	C	G	C	C	C	C
rs17069665	chr6:108941468	A=0.919, G=0.081	A	A	G	A	A	A	A
rs12213895	chr6:108951968	T=0.919, A=0.081	T	T	A	T	T	T	T
rs12212067	chr6:108981196	T=0.924, G=0.076	T	T	G	T	T	T	T
rs9398171	chr6:108983527	T=0.732, C=0.268	T	C	C	T	T	C	T
rs73763159	chr6:108991686	G=0.924, T=0.076	G	G	T	G	G	G	G
rs3800230	chr6:108998128	T=0.899, G=0.101	T	T	G	T	T	G	T
rs1935952	chr6:108998905	C=0.727, G=0.273	C	G	G	C	C	G	C
		Haplotype Count	120	31	15	13	6	5	5
		Haplotype Frequency	0.6061	0.1566	0.0758	0.0657	0.0303	0.0253	0.0253

Supplementary Figure 2. *FOXO3* longevity haplotypes. (**A**) Japanese Haplotype – allele frequency of haplotype = 0.10. (**B**) Caucasian haplotype – allele frequency of haplotype = 0.76. Tables of observed haplotypes were generated using LDHap (https://ldlink.nci.nih.gov/?tab=ldhap). Haplotypes with frequencies greater than 1% are displayed vertically and ordered by observed frequency in the selected query sub-population. Variant bi-allelic genotypes and frequencies are reported in rows and are sorted by genomic position. Links are available to dbSNP (https://www.ncbi.nlm.nih.gov/snp/) RS numbers and coordinates in the UCSC Genome Browser (http://genome.ucsc.edu/cgi-bin/hgGateway; version GRCh37 is shown). Reference populations were "JPN" Japanese, shown in "A", and "CEU" Caucasian, shown in "B", using data from Phase 3 (Version 5) of the 1000 Genomes Project (https://www.internationalgenome.org). Rectangles highlight the longevity haplotype (minor alleles noted in Supplementary Figure 1) that has a frequency of 0.101 in the Japanese population and 0.758 in Caucasians.
