# Supplementary material for: FOXO3 longevity genotype mitigates the increased mortality risk in men with a cardiometabolic disease
Source: Aging (Albany NY). 2020 Dec 1;12(23):23509–24. doi: 10.18632/aging.202175 (PMC7762472; doi:10.18632/aging.202175)
Supplement: Supplementary Tables [file aging-12-202175-s003.pdf]

SUPPLEMENTARY TABLES

Supplementary Table 1. Age-adjusted characteristics of subjects at baseline for *FOXO rs2802292 TT* genotype and *G*-allele carriers.

Variable	*TT*	*GT/GG*	p
n	1901	1683	
Age (years)	77.0 ± 4.5	78.0 ± 4.7	0.0008
BMI (kg/m²)	23.5 ± 3.2	23.5 ± 3.1	0.81
Fasting plasma glucose (mg/dl)	112.3 ± 28.0	113.9 ± 31.0	0.090
Smoking (pack-years)	25.4 ± 33.5	27.0 ± 35.3	0.20
Alcohol intake (oz/mo)	18.7 ± 39.8	19.0 ± 41.4	0.83
Physical activity index	30.9 ± 4.7	30.9 ± 4.5	0.94
Depressive symptoms (%)	10.0	11.2	0.29
Stroke (%)	4.5	4.4	0.85
Cancer (%)	14.3	12.8	0.22
CHD (%)	22.3	18.7	0.009
Diabetes (%)	27.0	30.4	0.027
Hypertension (%)	53.1	54.1	0.55
CHD or diabetes or hypertension (%)	70.0	70.2	0.92

Values shown are age-adjusted mean ± SD for indirect measures and proportion for direct measurements.

Supplementary Table 2. Effects of minor alleles on transcription factor (TF) binding.

SNP	Result	TF	Biological Pathways	Tissue*
rs768023	abolish	FOXA2	stem cell maintenance, vascular epithelia, diabetes, energy response	Liver, Lung, Pancreas, Stomach
	abolish	HDAC	stem cell maintenance	Lymphoblasts, Colon, Esophagus, Ovary, Small Intesting, Spleen, Thyroid
rs1536057	abolish	NRF1	stem cell maintenance	Brain
	abolish	E2F	DNA damage response	Lymphoblasts
	create	POU5F1	stem cell maintenance	-
rs2253310	create	TFCP2L1	stem cell maintenance	Kidney, Salivary Gland, Skin, Thyroid
rs2802288	create	MYF	stem cell maintenance, muscle atrophy	Muscle
rs2764264	abolish	NKX3	stem cell maintenance	Salivary Gland, Prostate
rs2802292	create	HSF1	stress response	most tissues, not Brain
rs12202234	abolish	FOXA	stem cell maintenance, vascular epithelia, diabetes, energy response	Bladder, Breast, Prostate
rs17069665	abolish	MITF	stem cell maintenance	Cervix, Uterus
rs12213895	create	MEF2	stem cell maintenance	Artery, Lung, Skin
rs12212067	create	MZF1	stem cell maintenance	Brain, Lung, Ovary, Pituitary, Prostate, Thyroid, Uterus
rs9398171	create	NR2F1	stem cell maintenance	Bladder, Brain, Cervix, Fallopian Tube, Lung, Nerve, Ovary, Prostate, Uterus
	create	HNF4	diabetes, energy response,	Colon, Liver, Small Intestine
	abolish	ONECUT1	stem cell maintenance, diabetes, energy response	Liver, Pancreas
rs73763159	abolish	PRDM1	stem cell maintenance	Esophagus, Vagina
rs3800230	abolish	FOXP1	stem cell maintenance	-
rs1935952	abolish	MZF1	stem cell maintenance	Brain, Lung, Ovary, Pituitary, Prostate, Thyroid, Uterus

The 14 SNPs modify 19 TF binding sites shown below, along with their functions. SNP ID# is variant name from the database dbSNP, create/abolish refers to whether the minor allele creates or abolishes a TF binding site.
Significant variants were identified previously [Donlon, T.A., et al. FOXO3 longevity interactome on chromosome 6. Aging Cell. 2017; 16:1016-1025] using HaploReg (https://pubs.broadinstitute.org/mammals/haploreg/haploreg.php), which is a tool for exploring annotations of the noncoding genome at variants on haplotype blocks, such as candidate regulatory SNPs at disease-associated loci [Ward, L.D. & Kellis, M. HaploReg: a resource for exploring chromatin states, conservation, and regulatory motif alterations within sets of genetically linked variants. Nucleic Acids Res. 2012; 40 (Database issue):D930-934]. Expression data are from GTEX; * >100 TPM.
